# INTENSIVE TREATMENT OF LEG LYMPHEDEMA

**DOI:** 10.4103/0019-5154.62745

**Published:** 2010

**Authors:** José Maria Pereira de Godoy, Lina M O Azoubel, Maria de Fátima Guerreiro de Godoy

**Affiliations:** *From the Department of Cardiology and Cardiovascular Surgery and professor of the post graduation course of Medicine School of São Jose do Rio Preto-FAMERP-Brazil.*; 1 *Nutritionist of the Godoy Clinic and Professor of the Post Graduation Course on Rehabilitation of Lymphevenous in Medicine School in São José do Rio Preto-FAMERP-Brazil of São Jose do Rio Preto, Brazil.*; 2 *Occupational Therapist, Professor of the Post Graduation course on Lymphovenous Rehabilitation – FAMERP and Clinic Godoy, São José do Rio Preto, Research Capes-Brazil.*

**Keywords:** *Bandaging*, *exercises*, *lymph drainage*, *lymphedema*

## Abstract

**Background:**

Despite of all the problems caused by lymphedema, this disease continues to affect millions of people worldwide. Thus, the identification of the most efficacious forms of treatment is necessary.

**Aim:**

The aim of this study was to evaluate a novel intensive outpatient treatment for leg lymphedema.

**Methods:**

Twenty-three legs of 19 patients were evaluated in a prospective randomized study. The inclusion criteria were patients with Grade II and III lymphedema, where the difference, measured by volumetry, between the affected limb below the knee and the healthy limb was greater than 1.5 kg. Intensive treatment was carried out for 6- to 8-h sessions in the outpatient clinic. Analysis of variance was utilized for statistical analysis with an alpha error of 5% (*P*-value <0.05) being considered significant.

**Results:**

All limbs had significant reductions in size with the final mean loss being 81.1% of the volume of edema. The greatest losses occurred in the first week (*P*-value <0.001). Losses of more than 90% of the lymphedema occurred in 9 (39.13%) patients; losses of more than 80% in 13 (56.52%), losses of more than 70% in 17 (73.91%) and losses of more than 50% were recorded for 95.65% of the patients; only 1 patient lost less than 50% (37.9%) of the edema.

**Conclusion:**

The intensive treatment of lymphedema in the outpatient clinic can produce significant reductions in the volume of edema over a short period of time and can be recommended for any grade of lymphedema, in particular the more advanced degrees.

## Introduction

Lymphedema is a type of edema that occurs due to an abnormal accumulation of fluids and other substances in the tissues resulting from a failure of the lymphatic system associated with insufficiency of extralymphatic proteolysis of proteins in the cell interstice and the mobilization of macromolecules,[1] such as hyaluronic acid.[2]

Lymph drainage,[3,4] exercising,[5,6] hygienic precautions,[7,8] compression mechanisms,[9] and more recently the association of occupational activities,[10] and cervical stimulation[11,12] are recommended in the treatment of lymphedema. When lymphedema is established, the degree of dysfunction is great due to physical factors such as a decrease in joint mobility causing reductions in the amplitude of movements, the weight of the limb, pain, and disability to perform day-to-day tasks.[13] Treatments focusing on decreasing arm volume without addressing issues of pain may not result in improvements in activity, participation, or health-related quality of life.[14,15]

Despite of all the problems caused by lymphedema, this disease continues to affect millions of people worldwide. Thus, the identification of the most efficacious forms of treatment of this disease is necessary. The objective of this study was to evaluate a novel intensive lymphedema treatment in the outpatient clinic.

## Methods

Twenty-three legs of 5 male and 14 female patients with ages ranging between 27 and 75 years (mean age 49.1 years) were evaluated in a prospective quasi-randomized study. The diagnosis of lymphedema was clinical with confirmation by lymphoscintigraphy for some patients. Inclusion of patients was by order of arrival in the treatment center where individuals were invited to participate after the objectives of the study had been explained. The inclusion criterion was patients with Grade II and III lymphedema when the difference between legs below the knees, as evaluated by volumetry, was greater than 1.5 kg. Patients who were unable to perform volumetry of the limb due to its size and the size of the container, those with acute infections, and those with differences of less than 1.5 kg were excluded from the study.

Volumetry using the water displacement technique was performed for the lower leg before the start of treatment and weekly thereafter for the 4-week study period. For the evaluation, improvements in the difference between the healthy and affected limbs were considered or in cases of bilateral lymphedema, the total reduction at the end of the treatment period.

An intensive form of treatment was utilized with daily 6- to 8-h sessions in the outpatient clinic. Mechanical lymph drainage using the RAGodoy® apparatus[16] was performed for 6 to 8 h associated with the Godoy and Godoy cervical stimulation technique for 20 min per day[11,12] and manual lymph drainage utilizing the Godoy and Godoy technique[3,17] for 1-h session daily. With the exception of the session of manual lymph drainage, compression was applied using a home-made stocking made of a low-elasticity cotton-polyester material.[2,18] First the compression stocking was put on, and then mechanical lymph drainage was performed followed by cervical stimulation and finally manual lymph drainage.

Analysis of variance was used for statistical analysis with an alpha error of 5% (*P*-value <0.05) being considered significant. The study was assessed and approved by the Research Ethics Committee of the Medicine School in São José do Rio Preto (FAMERP).

## Results

**Figure 1:**
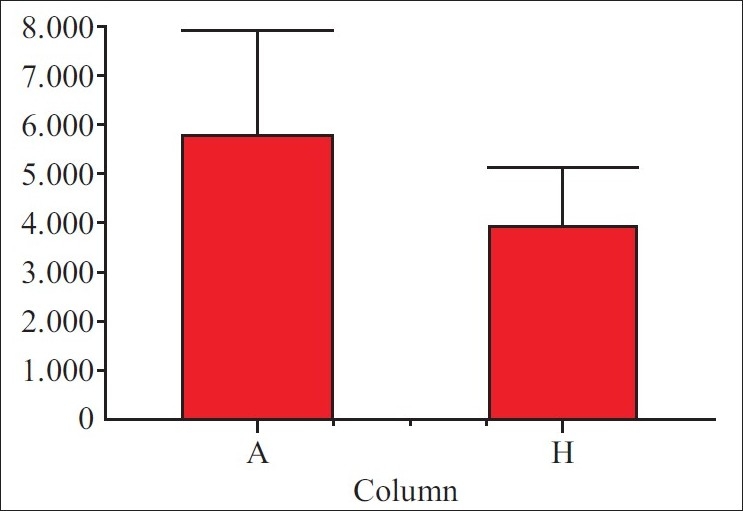
Mean and standard deviation of the total loss in volume of limbs between the start and the end of the 4 weeks of treatment

[Table 1] shows the percentage of losses in volume per week; all patients had a significant volumetric loss with the mean loss at the end of the study being 81.1% (*P*-value <0.001). Table 2 and Figure 1 show the mean and standard deviation of the total volume of the limb before and after 4 weeks. The greatest variations occurred in the first week (*P*-value <0.001), however, losses were seen throughout the entire treatment period [Table 1].

**Table 1:** Loss in volume (%) over the 4-week study

Lymphedema	Serial (N)	Week 1 (%)	Week 2 (%)	Week 3 (%)	Week 4 (%)
Unilateral	1	54.30	41.00	56.60	75.68
Bilateral	2	39.30	63.40	72.40	81
		23.10	58.2	70.70	81.10
Bilateral	3	20.09	42.34	50.00	53.84
		23.04	38.30	49.70	57.60
Unilateral	4	10.40	32.86	35.88	37.97
Unilateral	5	64	85.03	87.36	97.80
Unilateral	6	34.30	91.60	94.66	97.80
Unilateral	7	64.90	94.70	96.80	97.80
Bilateral	8	35.81	42.61	49.69	56.06
		27.74	51.33	51.87	54.87
Unilateral	9	72.20	86.30	91.00	98.70
Unilateral	10	52.50	54.43	61.14	70.30
Unilateral	11	45.67	58.16	67.55	79.24
Unilateral	12	56.12	75.13	77.50	98.17
Unilateral	13	61.13	67.25	80.65	91.17
Unilateral	14	62.62	79.77	87.93	97.01
Unilateral	15	42.45	59.69	68.40	70.49
Bilateral	16	49.39	56.40	57.62	84.84
		23.5	37	51	51.56
Unilateral	17	40.25	68.80	74.61	85.60
Unilateral	18	50.90	72.91	78.65	82.30
Unilateral	19	53.72	79.38	88.49	95.12

**Table 2:** Mean and standard deviation of the total loss in volume of limbs between the start and the end of the 4 weeks of treatment

Parameter	After	Before	Difference	*P*-value
Mean	5895.2	3983.0	1912.2	<0.001
# of points	23	23	23	
Standard deviation	2124.3	1248.0	2585.4	
Standard error	442.95	260.23	539.09	
Minimum	10.620	2035.0	-7670.4	
Maximum	9600.0	7681.0	5639.0	
Median	5431.0	3848.0	2138.0	
Lower 95% CI	4976.6	3443.3	794.13	
Upper 95% CI	813.9	4522.8	3030.3	

Volume losses of greater than 90% of the edema occurred in 9 (39.13%) patients, losses greater than 80% in 13 (56.52%), losses of more than 70% in 17 (76.91%), and losses greater than 50% were observed in 95.65% of the patients. Only one patient lost less than 50% (37.9%) of the volume of edema.

## Discussion

This study describes a novel intensive treatment for outpatients, which is efficacious in the reduction of lymphedema of the lower limbs. The advantage of this approach is the rapid reduction in volume in patients with the more advanced forms of the disease. Another advantage is the low cost of the compression stockings and the use of apparatuses to perform lymph drainage, which increases the tolerance of this intensive treatment.

To achieve this result, constant adjustments of the compression mechanisms are essential. Some patients have attained reductions of as much as 8 cm at the greatest circumference in a single day of treatment, and so these adjustments are essential to achieve large losses. Patients utilize compression stockings 24-h per day during this phase of the treatment. With the continuation of the treatment after this month, total reduction of the edema is possible in 90% of the patients.

Maintenance of the results is the main goal in lymphedema therapy where continuity of treatment utilizing an elastic compression or cotton-polyester stocking is necessary. Even so monthly evaluations of patients using volumetry are advisable to track volume changes; volumetry is the gold standard, it is a cheap examination and the physician can advise patients in cases of relapse and complications. Studies (in press) have shown that just with the use of a cotton-polyester stocking associated with controlled myolymphokinetic activities and exercises, it is possible to reduce the volume of edema. Self-lymphatic drainage can be performed by the majority of these patients as long as they are trained and followed up.[19] Hence, there are several options for both the treatment and maintenance of lymphedema. Another aspect to be considered is the clinical treatment of lymphedema, where surgery is only indicated for the removal of masses from the feet when necessary, a situation that occurred in two cases in this study. Control of the reduction of lymphedema of limbs is vital to avoid skin folds, as this can cause pain and skin lesions with to the use of compression. These complications during treatment were solved by reducing the time of treatment sessions, with careful observation until the skin retracted at which time compression could again be used. Thus, it is possible to reduce large volumes of lymphedema just with clinical treatment.

In this study we excluded patients with small differences between the legs because total reduction of the edema can occur within the first week for many of them and hence the criteria related to the weight difference were so that reductions could be observed during the entire 4-week treatment period.

Several observations were made during the evolution of this treatment which may help to better understand this approach to lymphedema treatment. One observation is related to the compression mechanism; its removal at bedtime allows an increase in the volume of the leg during the night and so for this reason the use of the stocking is necessary over the 24 h of this initial phase in order to achieve the greatest loss in volume. During the night, when patients remove the stockings the loss in volume will be slower. Tolerance is good, in part, because of the prospective of good results motivating the patients and hence contributing to treatment. In another study, we evaluated the association of elastic bandages utilized over the cotton-polyester stockings. This association increases the volume losses although the technique is not always tolerated well by patients.

The objective of these studies is to identify the most efficacious options in all forms of treatment including in respect to filariasis and children.[20,21]

Lymphoscintigraphy can be used for the diagnosis of lymphedema and to assess possible alterations that occur with the treatment.[22,23] The different forms of lymphedema treatment should be considered and adapted to the situation of each patient. This form of treatment can be adapted to treat large populations with lymphedema at a low cost and reduce the suffering of great numbers of patients with lymphedema.

## Conclusion

Intensive treatment of outpatients with all grades of lymphedema can give significant reductions in the volume of the edema over a short period of time particularly with the most advanced degrees of the disease.
